# Motif-Role-Fingerprints: The Building-Blocks of Motifs, Clustering-Coefficients and Transitivities in Directed Networks

**DOI:** 10.1371/journal.pone.0114503

**Published:** 2014-12-08

**Authors:** Mark D. McDonnell, Ömer Nebil Yaveroğlu, Brett A. Schmerl, Nicolangelo Iannella, Lawrence M. Ward

**Affiliations:** 1 Computational and Theoretical Neuroscience Laboratory, Institute for Telecommunications Research, University of South Australia, Mawson Lakes, South Australia, Australia; 2 California Institute of Telecommunications and Information Technology (Calit2), University of California Irvine, Irvine, California, United States of America; 3 Department of Psychology and Brain Research Centre, University of British Columbia, Vancouver, British Columbia, Canada; University of Michigan, United States of America

## Abstract

Complex networks are frequently characterized by metrics for which particular subgraphs are counted. One statistic from this category, which we refer to as *motif-role fingerprints*, differs from global subgraph counts in that the number of subgraphs in which each node participates is counted. As with global subgraph counts, it can be important to distinguish between motif-role fingerprints that are ‘structural’ (induced subgraphs) and ‘functional’ (partial subgraphs). Here we show mathematically that a vector of all functional motif-role fingerprints can readily be obtained from an arbitrary directed adjacency matrix, and then converted to structural motif-role fingerprints by multiplying that vector by a specific invertible conversion matrix. This result demonstrates that a unique structural motif-role fingerprint exists for any given functional motif-role fingerprint. We demonstrate a similar result for the cases of functional and structural motif-fingerprints without node roles, and global subgraph counts that form the basis of standard motif analysis. We also explicitly highlight that motif-role fingerprints are elemental to several popular metrics for quantifying the subgraph structure of directed complex networks, including motif distributions, directed clustering coefficient, and transitivity. The relationships between each of these metrics and motif-role fingerprints also suggest new subtypes of directed clustering coefficients and transitivities. Our results have potential utility in analyzing directed synaptic networks constructed from neuronal connectome data, such as in terms of centrality. Other potential applications include anomaly detection in networks, identification of similar networks and identification of similar nodes within networks. Matlab code for calculating all stated metrics following calculation of functional motif-role fingerprints is provided as S1 Matlab File.

## Introduction

Complex relational systems from different domains, such as biology, sociology or economics, can be systematically analyzed using their network representations. A *network* (also known as a *graph*) is composed of nodes and edges, where *nodes* represent the entities in the system and *edges* represent the relationships between these entities. Depending on the type of represented relations, the node pairs that form the edges can have a certain ordering, in which case the resulting network is called *directed*. For example, in networks of biological neurons and synapses (also known as *neuronal connectomes* [1]), the nodes correspond to individual neurons, while directed edges between the nodes (typically) represent the existence of chemical synapses that enable communications between neurons [2]. The wiring patterns of networks cast light on the functional mechanisms of the analyzed complex systems, and therefore, network structure analysis is gaining increasing interest from different disciplines.

However, many network analysis problems are computationally intractable [3]. Therefore, the only available solutions are based on approximations to the exact solutions of these problems. *Network properties* that describe different wiring characteristics of networks are used for this purpose. For example, given two networks without any labeling on the nodes, the problem of finding all the node pairs that have identical wiring patterns in the two networks is a computationally intractable problem. However, this problem can be simplified by computing the *degrees* (i.e., the number of neighbors a node has) of all nodes and using the degree statistics to compare the nodes. Even if the resulting matches are not guaranteed to have identical wiring patterns, these matches would extensively reduce the size of the search space. The search space can be reduced even further by computing other network properties that capture different types of interaction patterns; e.g., using the similarities of *clustering coefficients* that measure the tendency of nodes to form triangular interactions [4].

Different *subgraphs* of a network can be obtained from different subsets of its nodes and edges. Many of the network properties are indeed dependent on the subgraph properties of the networks; e.g., clustering coefficient is defined based on three-node subgraphs of a network in which all nodes are connected with each other forming a triangle. In a *connected* subgraph, all nodes are reachable from any of the other nodes in the subgraph. A subgraph is *induced* (also known as *node induced*) if it is enforced that all the edges between the chosen subset of nodes are included in the subgraph. The subgraphs that do not carry the induced property are called *partial* (also known as *edge induced*) subgraphs. For example, a 3-node *clique* contains 3 different two-path subgraphs (two-path subgraphs are those that contain 3 nodes and 2 edges) when partial subgraph properties are considered. However, such a graph does not contain any two-path subgraphs when induced subgraph properties are considered.

**Figure 1:**
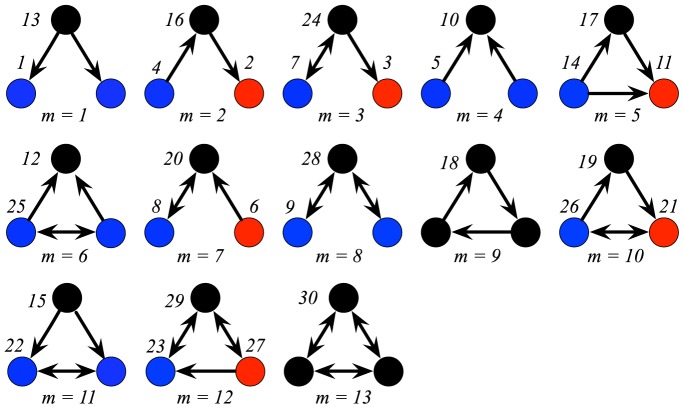
All 13 three-node connected motifs and all 30 three-node connected motif-roles. A directed network is assumed. The numerical label for each motif (denoted with the label *m*) is identical to that used in [9]. Each distinct motif-role within each motif is denoted by different colours, and the numerical label next to each node. The numerical label provided for each motif-role is represented by the label 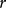 in the text and in Fig. 2, where 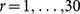.

**Figure 2:**
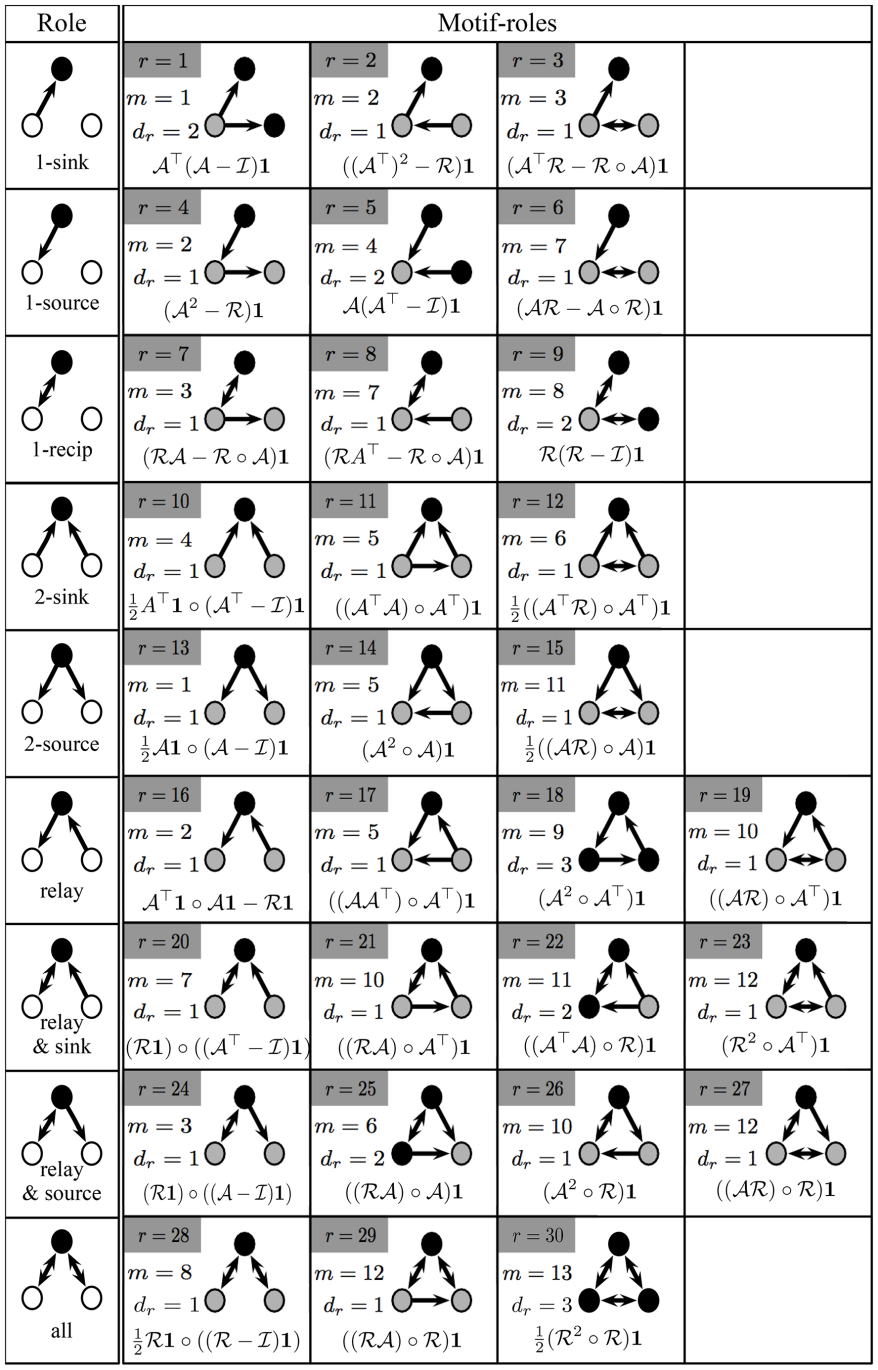
Formulae for counting the three-node motif-role fingerprints. The first column depicts the 9 distinct roles on functional motifs. Each row shows each three-node motif in which the corresponding role appears (indexed by 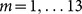), and the plurality 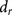 with which motif-role 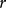 appears within motif 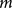 (see Methods). Black filled circles indicate the nodes in motif 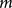 that play motif-role 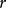 (see also Fig. 1). The equations shown for each role, *r*, are the entries of the functional motif-role fingerprint matrix, 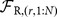, where 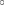 denotes the Hadamard product, 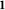 is an 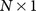 unit column matrix, 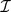 is the 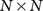 identity matrix, and 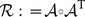 is the matrix of reciprocal edges.

Triangular patterns in networks are commonly utilized to analyze the network topology. In undirected networks, the *clustering coefficient* of a node is calculated by dividing the number of triangles around the node by the number of different pairs of its neighbors [5]. *Average clustering coefficient* explains the clustering (triangulation) within a network by averaging the clustering coefficients of all its nodes. Extension of clustering coefficient to directed networks is not trivial since there are two different types of triangular directed subgraphs; one being a cyclic subgraph (m = 5 in Figs. 1 and 2) and the other being an acyclic subgraph (m = 9 in Figs. 1 and 2). Based on the counts of the four distinct node roles on these two subgraphs (i.e., 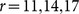 and 18 in Figs. 1 and 2), the definition of clustering coefficient has been extended to the directed case [4], [6]. A different metric for quantifying network clustering known as *transitivity* is calculated by considering every possible combination of three nodes in a network, and counting how many of these triads are mutually connected by three edges, normalized by the number of triads with at least two edges [7]. It is similar to clustering coefficient but unlike that metric, it is not an average of local node-specific clustering. Transitivity is typically used for undirected networks rather than directed ones, but an expression for directed transitivity is given in [8].

Recent work on network properties use the statistics of all observable connected subgraph configurations as detailed descriptors of the wiring in networks [9], [10]. *Network motifs* were originally defined as the partial subgraph patterns of a network that appears more frequently than expected from a ’null-hypothesis’ network model that preserve the input network's degree distribution, or other statistical properties [9], [11]–[15]. Network motifs are defined for both directed and undirected networks, covering all observable subgraphs patterns on sets of nodes ranging in dimension from 2 to *n*. Network motifs have been used to analyze network structures of a wide-range of networks, such as those of the neuronal connectome of *C. elegans* [16]–[20]. Practically, network motif analyses are performed with 3-node subgraph patterns due to the high computational cost of null model generation step for larger subgraphs; all directed 3-node subgraph patterns are illustrated in Fig. 1.

Another group of network properties that are based on subgraph counts have been studied in the context of *graphlets*—these are small, connected, non-isomorphic and induced subgraphs of a large network [10]. There are three major differences between network motifs and graphlets:

1. network motifs account for partial subgraphs while graphlets are based on induced subgraphs;
2. network motifs are dependent on a given null network model while graphlets are completely independent from any null hypotheses; and
3. graphlets are defined only for undirected graphs while network motifs are defined also for directed graphs.

The number of times that each graphlet appears in a network describes the network's topology [10]. Currently, the most advanced method for describing the topology of an undirected network is based on the dependencies between different graphlets [21].

Subgraph properties are not only useful for describing the topology of networks, but they can also be utilized for describing the local wiring around nodes. For instance, degree describes the wiring around a node by counting the number of edges touching the node. Replacing edges with subgraphs of each kind in this definition, the local wiring around a node can be described by the number of subgraph patterns that the node participates in. While these subgraph statistics on nodes can be computed without imposing any orientations on the subgraphs [8], [22], a node's *role* in the network can be characterized more accurately by introducing such orientation constraints based on the symmetries within the subgraphs [23]–[25]. For example, as illustrated in Figure 1 of [23], and Fig. 1 here, there are 30 unique motif-roles on the 3-node directed subgraph configurations. Przulj [25] identifies the *orbits* (i.e., the nodes that have identical wiring patterns within graphlets) of all 2- to 5-node graphlets and uses these orbits to describe the wiring around a node by defining *graphlet degree*, which is the number of graphlets that touch a node at an orbit. Furthermore, the vector containing the graphlet degrees of all 73 orbits of 2- to 5-node graphlets is termed the *graphlet degree vector* and successfully applied for identifying the wiring similarities between the nodes of a network, and also, between the nodes of different networks [26], [27]. It has been argued that analysis of neuronal connectome data will need to take into account node-referenced heterogeneity [28]–[30], such as measured by graphlet degree. Another possible application is in the analysis of genetic networks [31].

The terminology on subgraph properties is not well-defined, with some studies using the terms “subgraphs”, “network motifs” and “graphlets” interchangeably. In order to avoid confusion, we use the term “*functional motifs*” to represent the partial subgraph properties (e.g., network motif properties defined in [9]), and “*structural motifs*” to represent the induced subgraph properties (e.g., graphlet properties defined in [10], [25]) in a consistent manner with [8]. Structural motifs quantify anatomical building blocks, whereas functional motifs represent elementary processing modes of the networks [22]. This distinction between structural and functional subgraph properties have different implications for neuronal networks: structural motifs describe all synapses amongst a specific subset of neurons. In contrast, functional motifs can describe, for example, potential patterns of actual synaptic activations occurring (near) simultaneously amongst a specific subset of neurons. It is expected to observe correlation between structural and functional subgraph properties to some extent. Even though this is the case, the wiring characteristics that can be captured by these two types of subgraphs differ. For example, a node's importance in the networks as a ‘broker’ (e.g., 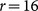 in Fig. 2) can only be captured by structural motifs since functional motifs consider also the cases that the node appears as roles 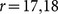 or 19 (Fig. 2). In these cases, the reference node is not a broker because of the edge between the two other nodes.

For both structural and functional motifs, we consider four different types of subgraph frequency derived network properties, as follows:

- **Global Metrics**: These metrics aim to describe the topology of an entire network.
**Motif Counts**: A network's topology can be described by the number of subgraphs that appear in the network. We use the term *motif counts* to represent these networks statistics. Different from the original definition of network motifs [9] (but consistent with usage in [8]), our motif statistics are independent of any comparison to null-hypothesis network model. For a given network, the corresponding motif counts form a *M* dimensional vector, each value representing the count for one of the *M* subgraphs.
**Motif-Role Counts**: A network's topology can also be described in terms of the roles within subgraphs. We use the term *motif-role counts* to represent the number of times that a given motif role appears in a network. Motif-role counts can be directly obtained by scaling the motif counts depending on the number of times the motif-role appears within the corresponding subgraph. For a given network, the corresponding motif-role counts form an *L* dimensional vector, each value representing the number of times one of the *L* node roles appears in the network.
- **Node-referenced Metrics**: These metrics aim to describe the local topology around a node in the network.
**Motif Fingerprints**: The wiring around a node in a network can be described by the number of subgraph patterns that it participates in, independent of the position (i.e., the role) on these subgraphs. Such statistics have been termed *motif fingerprints* [8], [22]. For each of the *N* nodes in a given network, the corresponding motif fingerprints are *M* dimensional vectors, each value corresponding to count of one of the *M* subgraphs that the node participates in.
**Motif-Role Fingerprints**: The wiring around a node in the network can be described at a finer detail by the number of subgraphs that touches the node at a specific orientation (i.e., on a node-role within the subgraph). We term such statistics as *motif-role fingerprints*. For each of the *N* nodes in a given network, the corresponding motif-role fingerprints are *L* dimensional vectors, each value corresponding to the number of subgraphs that touches a node at one of the *L* node-role positions.

**Figure 3:**
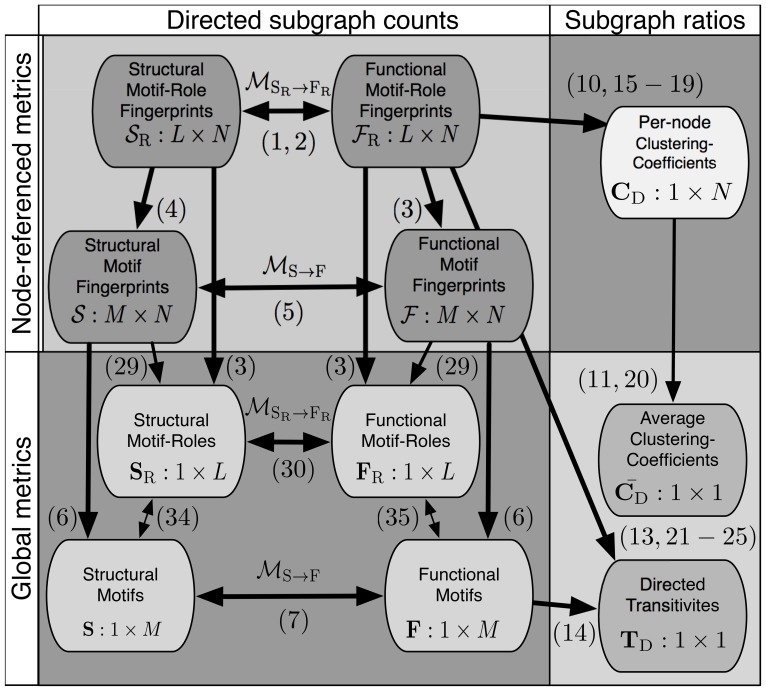
Dependencies between metrics that count three-node directed subgraphs. Arrows indicate that metrics can be derived from other metrics and numbers in brackets refer to equations in the text that mathematically describe these dependencies. The left side of the figure lists metrics that count subgraphs, while the right side shows metrics that are ratios of subgraph counts. The top half of the figure shows metrics that are node-referenced subgraph counts, while the bottom half shows metrics that are global subgraph counts.

In this study, we explore the relationships between all these different types of subgraph statistics (see Fig. 3). First, we present efficient ways of calculating the functional motif-role fingerprints of a given directed network. Second, we show that structural motif statistics can be derived from functional motif statistics and vice versa. This transformation enables efficient computation of structural motif-fingerprints which are computationally more expensive to obtain. Third, we show that the motif-role fingerprints are the most fundamental and informative of all the other subgraph metrics. We identify the transformations that derive all other subgraph statistics (i.e., motif fingerprints, motif-role counts, motif counts) from the motif-role fingerprints. Fourth, we discuss the relationships between motif-role fingerprints and directed clustering coefficients and transitivities, and show how these can be derived from motif-role fingerprints. Finally, we illustrate applications of these transformations on the neuronal connectome of *c. elegans*.

## Results and Discussion

While exploring the relationships between different subgraph properties, we assume a directed network with *N* nodes. The *adjacency matrix* representation of a network (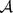) is an 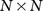 matrix, where 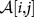 is 1 when there exists a directed edge from node 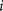 to node *j*, and otherwise 0. We label each of the 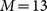 connected three-node motifs with the index 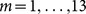 according to the classification introduced by [9]—see Fig. 1. When structural motifs of a directed network are considered, there are 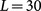 different motif-roles, which we label with the index 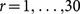, as illustrated in Fig. 1. However, when considering the functional motifs, these 30 motif-roles induces on 9 distinct roles—see Fig. 2. The ordering of our labels is determined by these roles, and hence is non-sequential when depicted in Fig. 1.

### Calculating Functional Motif-Role Fingerprints

We introduce two 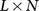 matrices, 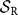 and 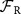, where the elements of the *i*–th column of these matrices is the transpose of the 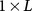 vector that denotes the structural motif-role fingerprints and functional motif-role fingerprints, respectively, in which node *i* participates. Fig. 2 lists equations that can be used to efficiently obtain all elements of the matrix 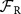, in terms of the adjacency matrix, 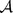. Further explanation on the computation of functional motif-role fingerprints is provided in the Methods section.

### The Relationship Between Structural and Functional Motif-Role Fingerprints

**Figure 4:**
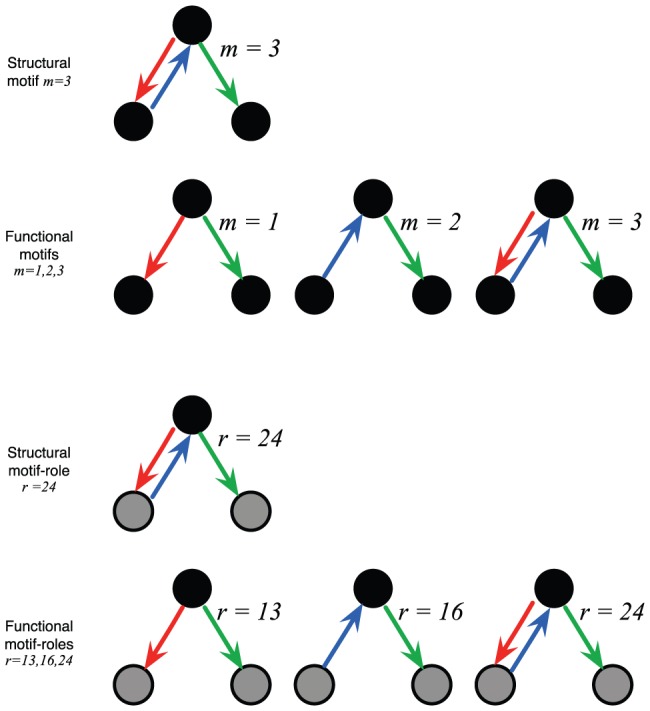
Structural motifs and motif-roles decompose into functional motifs and motif-roles. Illustration of the difference between structural and functional motifs and motif-roles. When counting structural motifs in a network, the connectivity between each set of three nodes is considered. In this case, if the nodes form motif 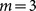, then this counts as one instance of structural motif 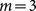, and no instances of structural motifs 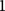 or 2. However, the same subgraph provides one instance each of functional motifs 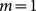, 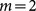, and 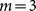 (see also Fig. 1 in [22] for a similar illustration). Consequently, there are no more structural motifs in total than the number of combinations of three nodes. However, this is not the case for functional motifs, since the same set of three nodes can contain multiple functional motifs. The same decomposition occurs for motif-roles. In the example in this figure, a single instance of structural motif-role 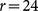 decomposes into one instance each of functional motif-roles 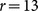, 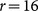 and 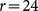.

Structural motifs (as counted for an overall network) can contain multiple functional motifs as illustrated in Fig. 4. We extend the distinction between structural and functional motifs, and show that the motif-role fingerprints of these two types of motifs can be derived from each other.

The mathematical relationship between structural and functional motif-role fingerprints can be conveniently expressed as
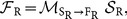
(1)where 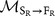 is an invertible 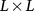 upper-triangular matrix, in which element 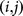 indicates how many copies of functional motif-role 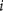 are contained in structural motif-role 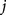 (see Equation (27) in Methods).

The fact that this matrix is invertible is important for numerical calculation of structural motif-role fingerprints. Although expressions for functional motif-role fingerprints can be efficiently calculated (see above and Fig. 2), it is more difficult to derive simple expressions for structural motif-role fingerprints. Instead, the inverse relationship
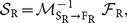
(2)where 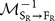 is given by Equation (28) in Methods, enables the structural motif-role fingerprint vector to be obtained without directly using the adjacency matrix. Moreover, the fact that 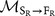 is invertible means that a unique structural motif-role fingerprint vector exists for any given functional motif-role fingerprint vector.

### Motif-Fingerprints and Global Motif Counts from Motif-Role Fingerprints

We now introduce the *motif-fingerprint* matrices, 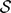 and 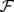, each of size 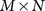, where the elements of the *i*–th column of these matrices denote the total number of structural motifs and functional motifs respectively in which node *i* participates [22]. The entries in the motif-fingerprints matrix can be trivially obtained from the motif-role fingerprints as follows:
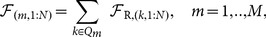
(3)

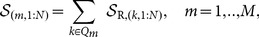
(4)where 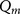 is the set of motif-role indices corresponding to motif index *m*. These sets can be readily identified in Fig. 1. The relationship between structural and functional motif fingerprints can be expressed as
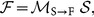
(5)where 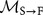 is a 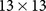 upper-diagonal invertible matrix in which element 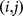 indicates how many copies of functional motif 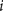 are contained in structural motif 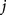 (see Equation (31) in Methods).

Various methods exist for obtaining motif counts within networks, as reviewed by [32]. Here, we state how such counts for three-node motifs can be calculated from motif fingerprints. We introduce the length 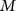 vectors **S** and **F**, where the elements of each vector (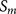 and 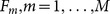) denote the total number of structural motifs and functional motifs, respectively. Obtaining the global motif counts from the motif fingerprints is a simple matter of summing the fingerprints for all nodes, and dividing by three, since each global motif appears in the fingerprint of exactly three nodes:
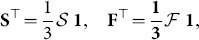
(6)where we also have



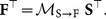
(7)Similarly to motif-role fingerprints, the existence of an invertible matrix for converting between functional and structural motifs implies that a unique structural motif or motif-fingerprint vector exists for any given functional motif or motif fingerprint vector.

### Directed Clustering Coefficients & Transitivities from Motif-Role Fingerprints

We now consider directed clustering coefficients and directed transitivities, and demonstrate how they are simple derivatives of motif-role fingerprints. We begin by defining two length 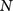 vectors; the first is the total number of closed directed triangles in which each node participates,
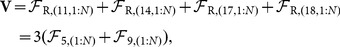
(8)and the second is the total number of potential triangles in which each node may participate,



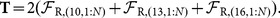
(9)The total directed clustering coefficient per node as derived by [4] may be expressed as the 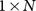 vector
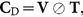
(10)where 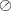 indicates Hadamard division. In any instance where division by zero occurs, we set the corresponding term of the result vector to zero. Because 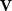 cannot be written in terms of functional motif fingerprints (since roles are integral to the definition of the various directed clustering coefficients), it is clear that finding specific functional motif-roles is a necessary step in finding the directed clustering coefficient. The global mean directed clustering coefficient is



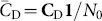
(11)

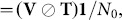
(12)where 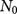 is the count of all nodes 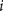 for which 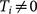.

The transitivity of an undirected network is defined as the ratio of the total number of three-node subgraphs with three edges, to one third of the total number of pairs of edges that share a node [7]. Consequently, transitivity measures the fraction of potential closed ‘triangles’ in a network that actually do form closed triangles.

Generalization to a definition of *directed transitivity* was given by [8]. This can be re-expressed in terms of elements from the functional motif-role matrices as
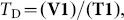
(13)or, unlike 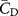, in terms of functional motif counts as



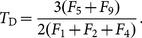
(14)In addition to the total directed clustering coefficient, [4] also described four sub-types of directed clustering coefficient, both on a per-node basis and as a global network average (see also [6]). These arise from the four motif-roles that exist within the two closed-triangle motifs with no reciprocal edges, i.e. motifs 5 and 9. In [4] these four types are referred to as ‘in’,‘out’, ‘middleman’ and ‘cycle’. Here we express these subtype clustering coefficients in terms of motif-role fingerprint vectors as
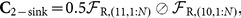
(15)

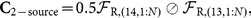
(16)

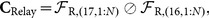
(17)

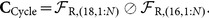
(18)


The factors of 0.5 arise from the two possible edges that can be added to motif-roles 10 and 13 to form closed directed feed-forward triangles.

We note that a comparison of the relative abundance of specific functional motif-role fingerprints for nodes of a given degree, with those in an in- or out-degree-preserving null-hypothesis network is equivalent to a comparison between elements of **C** vectors in the two networks. This is because a degree-preserving null-hypothesis network ensures that counts of motif-roles 10, 13 and 16 do not change. On the other hand, the utility of per-node clustering coefficients is that normalisation enables comparisons between nodes with different degrees within the original network. The situation is different for structural motif-roles; a null-hypothesis network will not have the same counts of structural motif-roles 10, 13 and 16 as the original network, which suggests there is possible utility in defining directed *structural* clustering coefficients, as alternatives to those of [4].

This discussion also suggests that additional sub-type directed clustering coefficients could be of interest. For example, the *3-feedforward clustering coefficient*:
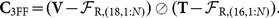
(19)


The global mean directed clustering coefficients are trivially obtained in the same way as the global mean directed clustering coefficient, i.e,
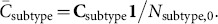
(20)


The different subtypes of clustering coefficient introduced by [4] suggest analogous forms of directed transitivity:
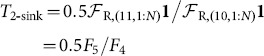
(21)

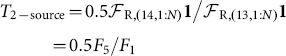
(22)

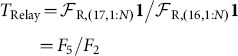
(23)

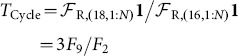
(24)

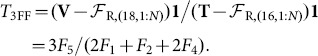
(25)


In the first of the two equations for *cycle transitivity*, we have been able to arbitrarily choose one of the three roles for motif 2 in the denominator, since when summed over all *N*, the results are identical for all three roles. The last expression, for *3-feedforward transitivity*, quantifies the total fraction of possible non-cyclic directed closed triangles that exist in a network.

### Remarks on Undirected Networks

The transitivity of a directed network without regard to the direction of the edges could potentially be of interest. Given that 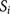 is the number of structural motif counts of type 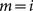, let 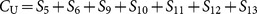. The undirected transitivity can be written as
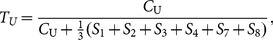
(26)where 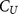 is the total number of closed triangles in a network written in terms of structural motifs counts. This result is equivalent to that of the standard definition of transitivity for an undirected network [7], [33], if the directed adjacency matrix was converted to undirected.

### Examples: Application to analysis of the *C. elegans* neuronal connectome

As an example application, we calculated the structural and functional motif-role fingerprints for the *C. elegans* hermaphrodite and male neuronal networks. The results are shown in Table 1, which enumerates the motif role fingerprints for neuron AVAR in the hermaphrodite.

**Table 1:** Example of structural and functional motif-role fingerprints: Neuron AVAR (node id 56) in the *C. elegans* neuronal network.

Role, *i*	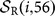	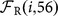
1	127	603
2	157	593
3	36	105
4	57	337
5	39	493
6	18	63
7	54	227
8	124	291
9	3	39
10	561	1176
11	62	254
12	7	31
13	615	1176
14	14	172
15	1	13
16	1258	2388
17	27	285
18	8	90
19	3	39
20	352	624
21	9	71
22	94	156
23	13	35
24	362	624
25	98	148
26	7	57
27	1	23
28	40	78
29	27	49
30	11	11

As mentioned, it is straightforward to derive the global subgraph ratio metrics (i.e., average directed clustering coefficients and directed transitivities) from motif-role fingerprints, as indicated in the bottom right part of Fig. 3. As described above, consideration of motif-role fingerprints led us to define six directed transitivities and six directed average clustering coefficients.

**Figure 5:**
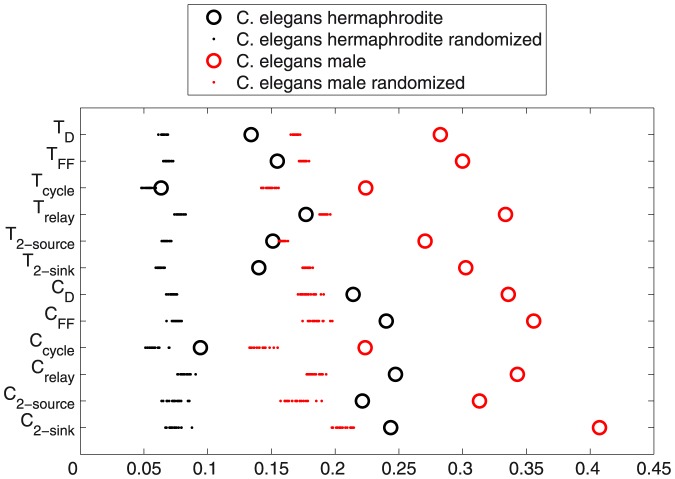
Directed transitivities and average clustering coefficients for two directed *C. elegans* chemical synapse networks, and randomisations of those networks. Circles show each of the six directed transitivities and six directed clustering coefficient values for the *C. elegans* hermaphrodite and male networks. Dots show comparison points obtained from each of 20 degree-preserving randomizations of the two connectivity matrices. Clearly the male exhibits higher transitivity and clustering than the hermaphrodite, according to all 12 statistics, but both real networks are more transitive/clustered than corresponding null-hypothesis networks.

Fig. 5 compares each of these transitivities and clustering coefficients for the two *C. elegans* neuronal networks, with those that result from in and out degree-preserving randomization of the *C. elegans* connectivity matrix. In each case, 20 randomized networks were created (we found that this was many more than were necessary to obtain consistent and significant changes in all metrics), and their transitivities and average clustering coefficients are plotted. Our value of 0.22 for the directed clustering coefficient of the source role (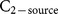) is consistent with result published in [17], as is our value for the corresponding randomized network of 0.076) but none of the other directed clustering coefficients were mentioned in [17].

We observe that the *C. elegans* hermaphrodite chemical synapse network is between 1.2 and 3.3 times more transitive or clustered (depending on the specific metric) than degree-preserving randomizations of the network (ratios were calculated with respect to the mean of the statistics for all network randomizations). This result is consistent with previous evaluations of clustering coefficient for this network (e.g., [17]). It is also clear, however, that among all the metrics, cycles have the smallest ratio, for both directed transitivity and average directed clustering coefficient. This is also consistent with prior analysis, such as that obtained via standard directed motif analysis — see Figure 7 in [17]. We also found that the male has higher ratios than the hermaphrodite, ranging from 2.1 to 3.9 times more transitive or clustered than the corresponding null hypothesis networks.

It is potentially of interest (both for *C. elegans*, and any other neuronal network data) to consider whether functional significance can be inferred from this form of analysis of directed clustering coefficient and transitivities. We expect, however, that analysis of motif-role fingerprints will likely be more revealing.

Next, we aim to identify particular network nodes that participate in an overabundance of some specific role, compared to a randomized network.

A simple example that illustrates the utility of obtaining motif-role-fingerprints is as follows. For the *C. elegans* hermaphrodite, we obtained 20 randomized networks, and identified the individual neuron that participated in the greatest number of each of the 30 roles, above the mean obtained in the randomized network. For many of the roles, we observed that the highly ranked neuron according to this metric had a high in and/or out degree. So next, we scaled by the total degree (i.e., in plus out degree) of each neuron, and examined the neurons with the highest ratios.

In this manner, we observed that neuron RIAL participates in 234 separate instances of functional motif-role 20, whereas in the corresponding randomized networks, RIAL on average participated in 53.3 instances of functional motif-role 20. This can be explained statistically, since RIAL participates in 9 reciprocal edge pairs to and from other neurons, and the our randomization algorithm does not preserve reciprocal degree, only in and out-degree.

A case of a neuron participating in an overabundance of a role that does not include reciprocal edges is that of neuron FLPR, and role 14. In the *C. elegans* network, FLPR participates in 80 instances of functional motif-role 14. The mean number of participations in the randomized networks, however, is only 14.75. Since motif-role 14 involves two outward edges from the reference node, and an edge between the two destination nodes, the motif-role analysis suggests that a role of neuron FLPR is to influence pairs of nodes that are themselves connected.

These few examples illustrate one of the potential applications for motif-role fingerprints: to identify interesting or anomalous nodes within a directed network so that further analysis or experimentation can be carried out on that node or its neighbors.

### Future Extensions and Applications

In order to account for heterogeneity in network structure and node types, we have derived mathematical relationships that we expect to be useful when motif distributions need to be characterised, either structurally or functionally, on a node-participation basis, rather than relative to the entire network. We have demonstrated that a hierarchy of relevant metrics exist, with summary metrics such as transitivity derived from richer and more informative vector statistics. The dependencies between each metric discussed are summarized in Fig. 3. We now discuss some anticipated applications and extensions of this work.

#### Analysis of Neuronal Connectome Data and Synaptic Polarities

Although the neuronal network of the nematode worm, *C. elegans*, is the only complete neuronal network obtained to date [17], network analysis will soon be required for the large neuronal network data sets that result from new experimental techniques currently under rapid development [28], [34]. Indeed, new methods have already resulted in a second partial neuronal network for the *C. elegans* male [19], and we used resulting network data in this paper.

In previous work on motifs applied to neuronal networks, it was observed that combining topological data with data on the functional role of neurons in *C. elegans* (sensory, motor or interneuron) allows a richer analysis of motif distributions with greater relevance to understanding than does describing structural motifs alone [18]. Both the work of [18], and the analysis of motifs in [16], [17], [22], however, characterized the hermaphrodite *C. elegans* neuronal network only in terms of overall abundance of each kind of motif, and did not study the number of motifs of each kind in which individual neurons participate. This is also the case for the analysis of the male posterior neuronal network reported by [19]. One possible direction is to use motif-roles to quantify the centrality of particular neurons within a network, such as by extending the work of [35] to take roles into account.

We anticipate that sophisticated analyses of directed complex neuronal network in future will make use of node-referenced role information, such as that provided by motif-role fingerprints discussed in this paper. Analysis of topological roles in neuronal connectome data could also be supplemented by physiological information, such as the polarity (excitatory or inhibitory) of synapses [30]. This could be modelled as signed edges, and motif-roles generalised to *Signed-motif-roles*.

#### Subgraphs with More Than Three Nodes

We note that the concept of motif-role fingerprints, either functional or structural, can be extended to arbitrary numbers of nodes per subgraph. For motifs with more than three nodes, however, the number of motif-role types becomes very large, which means that obtaining expressions for each element of 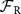 is more difficult. For example, it is known that for four-node subgraphs, there are 199 different connected directed subgraphs. We have not counted how many unique roles there are within each of these, but obviously there are at most a total of 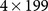 motif-roles for 4-node subgraphs. Calculation of 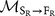 would also be tedious. Still, it need only be carried out once.

Although we leave this calculation for future work, we note that if this matrix was unknown, but alternative methods for finding both functional and structural motif-role fingerprint counts were available, then 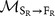 can readily be derived empirically using data from random directed networks. We have used this method to obtain the matrix 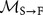 (and its inverse) for the case of 4-node global motifs. This was achieved using the Matlab software package known as the *Brain Connectivity Toolbox*, made available in association with [8], which provides code for obtaining global functional and structural motif counts for 4-node connected subgraphs.

#### Extension to Weighted Network Edges

The definition of motifs (in the global sense) has previously been extended to incorporate information about edge weights [36]. The resulting metric was referred to as *subgraph intensity*. It is potentially useful to extend this idea to motif-roles, and perhaps it will be as simple as replacing the binary adjacency matrix 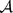 with a weighted adjacency matrix 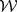 in the equations shown in Fig. 2. However, we leave consideration of this possibility for future work.

#### Possible use in role detection and detection of similar nodes and similar networks

There has been recent interest in automatic discovery of network roles, and nodes that are structurally similar, and algorithms have been developed for achieving this [37]. The methods described in [37] are flexible in the sense that many different network statistics can be provided as inputs from which roles are identified. There is strong potential for including motif-role fingerprints as a subset of the network statistics used in such algorithms. If, in the future, many large connectome datasets become available, it may be potentially interesting to assess the resulting networks for overall similarity, or to search for similar nodes within or across networks.

## Methods

### Source code

Matlab code implementing the results of this paper is provided as **S1 Matlab File**.

### Notation for Functional Motif-Role Fingerprints in Fig. 2

For a network with 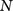 nodes, we denote the 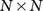 binary directed adjacency matrix as 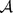 (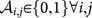). We assume that 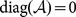, i.e there are no self-connections.

In the formulae listed in Fig. 2, we make use of the matrix 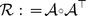, which is a binary matrix where each 1 indicates a reciprocal edge between two nodes. The symbol 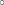 denotes the Hadamard (or Schmur) product, which is equivalent to term by term multiplication of two matrices of the same size.

Although some of the formulae can be rewritten in terms of the 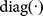 operator (e.g. 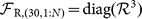), we have aimed to show that all elements of 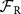 can be obtained with no more than two 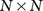 matrix multiplications and two Hadamard products, thus avoiding unnecessary multiplications.

Since there are three nodes in each motif, there can be no more than 3 role types for each motif; there are less in some instances where more than one node has the same role. Consequently, the figure also shows the number of nodes, 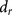, within each motif that play role *r*, and we have 
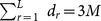
.

For completeness, we note that in our notation the matrix products 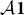 and 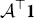 provide expressions for the out-degrees and in-degrees of each node, while 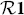 provides an expression for the total number of reciprocal edges in which each node participates. Also, we have 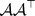 as the ‘Co-citation matrix’ [38] and 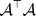 as the ‘bibliographic coupling matrix’ [38].

### Converting structural to functional motif-role fingerprints and *vice-versa*

The following 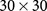 matrix enables conversion from structural motif-role fingerprints, 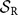 to functional motif-role fingerprints, 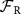, as expressed in Equation (1).
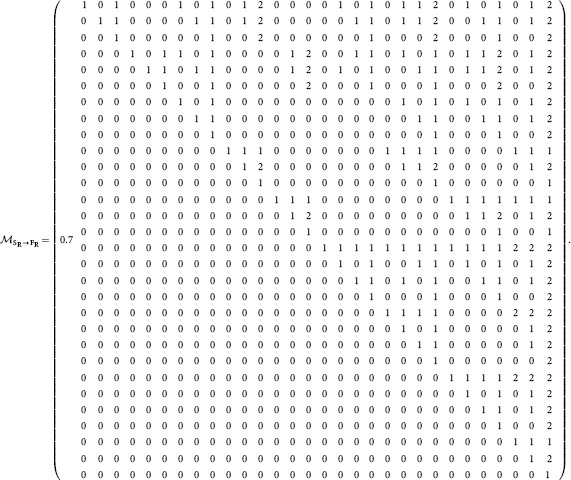
(27)


The following matrix is the inverse of 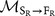, and can be used to convert from functional motif-role fingerprints, 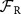, to structural motif-role fingerprints, 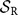, as expressed in Equation (2).
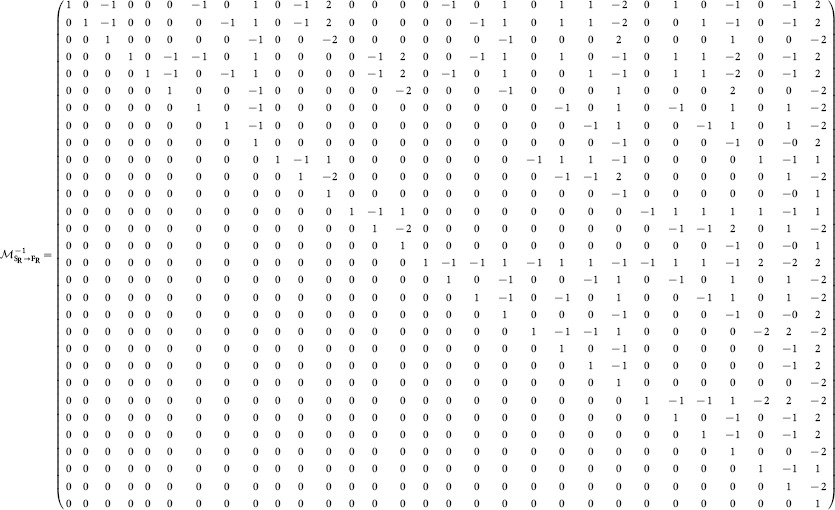
(28)


For completeness, as indicated in Fig. 3, we also introduce the *motif-role count* vectors 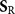 and 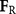, each of length *L*, where the elements of each vector (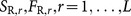) denote the total count of each structural motif-role and functional motif-role respectively, for an entire directed network. Obtaining the motif-role counts from the motif-role fingerprints is a simple matter of summing the fingerprints for all nodes, i.e.,
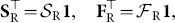
(29)where **1** is a column vector with all elements equal to unity. It is simple to show from Equation (1) that we also have



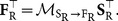
(30)


### Converting structural to functional motif fingerprints and *vice-versa*

The following matrix enables conversion from structural motif fingerprints, 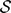 or structural motif counts, **S** to functional motif fingerprints, 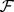 or functional motif counts **F**, as expressed in Equations (5) and (7) respectively.
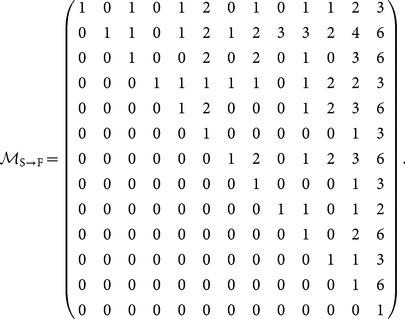
(31)


The following matrix is the inverse of 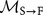, and can be used to convert from functional motif fingerprints, 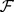, or functional motif counts, **F** to structural motif fingerprints, 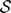 or structural motif counts, **S**.
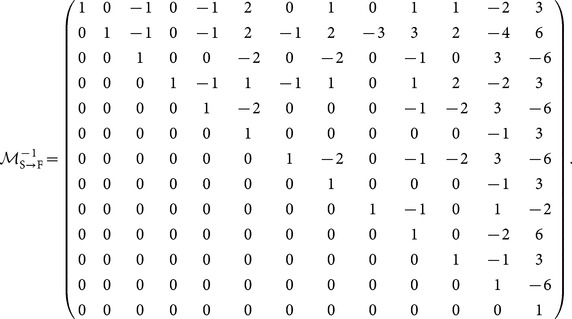
(32)


### Deriving motif counts from motif-role fingerprints

Given that each motif is comprised from three motif-roles, deriving the motif counts from the motif-role counts, or *vice-versa* is trivial. To make this relationship explicit, we introduce the following 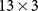 matrix composed from the elements of **F**_R_ (denoted as 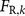, 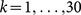) to explicitly denote which functional roles are associated with which functional motifs:
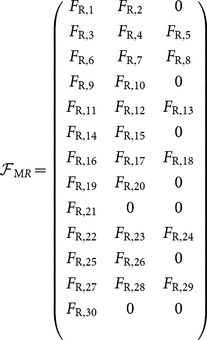
(33)


The *i*–th row in 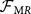 indicates motif 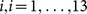. A zero appears for any motif in which more than one node plays the same role. Where 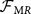 has three non-zero elements, they all have the same value, which is equal to the total number of functional motifs corresponding to that row. Where it has two elements, one element is twice the other, where the element multiplied by 2 is that indicated by 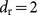 in Fig. 2. Similarly, where there is one element, it is multiplied by 3 as indicated by 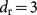 in Fig. 2.

We also introduce 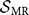 to denote the equivalent matrix for structural motifs. The total count of structural or functional motifs in a network can be trivially obtained from 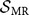 and 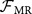 respectively by
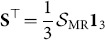
(34)

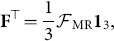
(35)where **1**_3_ is a unit 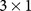 column matrix.

Conversely, the vectors **F**_R_ and **S**_R_ can be trivially obtained from **F** and **S** respectively, since we also have
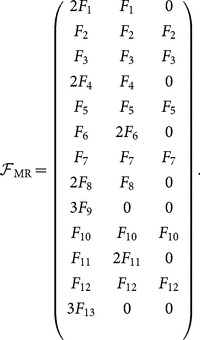
(36)


### Network data for *C. elegans* neuronal connectomes

For the hermaphrodite, we used network adjacency matrix data, based on chemical synapses, made publicly available in conjunction with [17]. For the male, we used network adjacency matrix data, based on chemical synapses, made publicly available in conjunction with [19].

## Supporting Information

S1 Matlab File **Matlab code implementing the results of this paper.**
(M) Click here for additional data file.
